# Layer-Structured Design and Fabrication of Cyanate Ester Nanocomposites for Excellent Electromagnetic Shielding with Absorption-Dominated Characteristic

**DOI:** 10.3390/polym10090933

**Published:** 2018-08-21

**Authors:** Fang Ren, Zheng-Zheng Guo, Han Guo, Li-Chuan Jia, Yu-Chen Zhao, Peng-Gang Ren, Ding-Xiang Yan

**Affiliations:** 1 The Faculty of Printing and Packaging Engineering, Xi’an University of Technology, Xi’an 710048, China; renfang0824@163.com (F.R.); 18829029028@163.com (Z.-Z.G.); 15129073264@163.com (H.G.); 2 College of Polymer Science and Engineering, State Key Laboratory of Polymer Materials Engineering, Sichuan University, Chengdu 610065, China; jialichuan@stu.scu.edu.cn; 3 School of Automation and Information Engineering, Xi’an University of Technology, Xi’an 710048, China; zhaoyuchen@xaut.edu.cn

**Keywords:** layer-structured, polymer composites, electromagnetic interference shielding, absorption-dominated

## Abstract

In this work, we propose novel layer-structured polymer composites (PCs) for manipulating the electromagnetic (EM) wave transport, which holds unique electromagnetic interference (EMI) shielding features. The as-prepared PCs with a multilayered structure exhibits significant improvement in overall EMI shielding effectiveness (EMI SE) by adjusting the contents and distribution of electrical and magnetic loss fillers. The layer-structured PCs with low nanofiller content (5 wt % graphene nanosheets (GNSs) and 15 wt % Fe_3_O_4_) and a thickness of only 2 mm exhibited ultrahigh electrical conductivity and excellent EMI SE, reaching up to 2000 S/m and 45.7 dB in the X-band, respectively. The increased EMI SE of the layer-structured PCs was mainly based on the improved absorption rather than the reflection of electromagnetic waves, which was attributed to the “absorb-reflect-reabsorb” process for the incident electromagnetic waves. This work may provide a simple and effective approach to achieve new EMI shielding materials, especially for absorption-dominated EMI shielding.

## 1. Introduction

With the unprecedented development of mobile phones, notebook computers, wireless routers, and other electronic devices, electromagnetic interference (EMI) shielding has become a form of silent pollution, which seriously affects the normal operation of other electronic circuits nearby and even endangers human life [1,2,3,4,5,6]. An effective method to solve the thorny problem is using EMI shielding materials to eliminate these unwanted electromagnetic energies.

Compared with traditional metallic EMI shielding materials, polymer composites (PCs) containing carbonous nanofillers have been viewed as advanced candidates for EMI shielding due to their lightweight, good processability, resistance to corrosion, and tunable electrical conductivity [7,8,9,10,11,12,13,14]. However, traditional PCs shielding materials with good EMI shielding property possess high conductivity of at least 1 S m^−1^ [15,16], resulting in excessive electromagnetic reflection because of the impedance mismatch. Thus, ideal EMI shielding materials with high shielding efficiency entailing strong electromagnetic wave absorption and low reflection have become an increasing requirement for the next-generation of communication technology and high-power electronic instruments.

For decades, researchers have made considerable efforts towards designing and fabricating various shielding materials by adjusting the dielectric constant and magnetic permeability in the pursuit of low interfacial impedance, as well as high loss ratio of incident microwave [2,17,18]. It has been reported that the combination of conductive fillers and magnetic nanoparticles can obviously improve the contribution of EM wave absorption to total shielding effectiveness. As well known, carbon materials, particularly graphene nanosheets (GNSs), have already been proven as good EM wave shielding materials with many unique properties, including ultrahigh conductivity, light weight, good corrosion resistance, high thermal stability, and high chemical stability [1,3,4,19,20]. In our previous research [21], we preliminarily proved that a synergistic effect exists between graphene and magnetic particles. These materials can prompt magnetic and electrical losses, and absorb electromagnetic waves more efficiently and mitigate the impact of secondary electromagnetic radiation to a considerable degree. Even so, for such materials to achieve a satisfactory EMI SE higher than 20 dB always require a high nanofiller loading and large material thickness, which seriously affects the cost, flowability, processing characteristics, and toughness of composites [22,23]. Thus, preparing PCs with superior EMI SE at low nanofiller loading remains a mission of shouldering heavy responsibilities. It is well known that regulating the structure of PCs has a remarkable influence on the electrical conductivity, which is directly related to EMI SE [24,25,26,27,28,29]. Recently, a large number of researchers have proven that PCs with layer-structured possessed superior EMI SE than conventional PCs at low filler content [20,26,30,31,32,33,34,35]. For example, composites prepared by stacking ten layers of 0.1 mm multiwalled carbon nanotubes/polymethyl methacrylate (MWCNT/PMMA) have a higher SE than that prepared from a single 1.0-mm-thick piece of bulk MWCNT/PMMA [30]. Besides, Pande et al. [35] reported seven layers of 0.3-mm-thick MWCNT-PMMA composite films with EMI SE up to 40 dB in the frequency range of 8.2–12.4 GHz, which is superior to stacking two layers of 1.1 mm thick MWCNT-PMMA bulk composite (30 dB). Additionally, such layer-structured composites result in the “absorb-reflect-reabsorb” process when electromagnetic waves penetrate, and therefore lead to the absorption dominant shielding mechanism [36,37,38]. Up to now, layer-structured PCs mainly focused on thermoplastic materials, the design and construction of layer-structured PCs in thermosetting resins remains a daunting challenge [26,31,39,40,41,42,43].

Cyanate ester (CE) is one of the most important high-performance thermosets, which can present excellent mechanical properties, outstanding heat, and good processing characteristics. In addition, CE has been widely used in aerospace and defense industries. Therefore, the composites based on CE have great potential to be utilized in various fields [44,45,46,47,48].

In this work, we fabricated a novel layer-structured CE-based composite with ultra-efficient EMI SE and low reflection characteristics via a simple mechanical mixing and hot-pressing method. The initial layer contains only Fe_3_O_4_ as the penetrating layer. The interlayer composed of Fe_3_O_4_ and GNSs serves as an efficient electromagnetic wave absorption layer that endows the composite with strong absorption ability. While the highly conductive GNS network at the bottom guarantees the composites have an excellent electromagnetic shielding ability. The layer-structured composite with a thickness of only 2 mm could reach an excellent EMI SE value of up to 45.7 dB in the X-band at the 5.0 wt % GNS and 15 wt % Fe_3_O_4_ loading. This result shows great promise for an ideal material with excellent EMI shielding in today’s portable electronic devices. In addition, the analysis of EMI shielding indicates that absorption is the primary shielding mechanism in the layer-structured nanocomposites due to “absorb-reflect-reabsorb” electromagnetic shielding mode.

## 2. Materials and Methods

### 2.1. Materials

Graphene nanosheets (average thickness < 30 nm, specific surface area ~ 60 m^2^ g^−1^) were supplied by Suzhou Kaier Graphene Co., Ltd. (Suzhou, China). Fe_3_O_4_ nanoparticles (12–15 nm) were provided by Chengdu Nuclear 857 New Materials Co., Ltd. (Chengdu, China). Bisphenol-A cyanate ester (CE) with molecular weight of 278.31 and melting point of 81.2 °C was purchased from Yangzhou Tianqi new material Co., Ltd. (Yangzhou, China). Bisphenol-A based epoxy (Epoxy E-51) with epoxide equivalent weight of 184–200 g per equiv. was purchased from Lanxing resin Co., Ltd. (Chengdu, China). All other reagents and solvents were purchased from Beijing Chemicals Factory (Beijing, China). All chemicals were of reagent grade and used without further purification.

### 2.2. Preparation of Layer-Structured GNSs/Fe_3_O_4_/CE Nanocomposite

**Figure 1:**
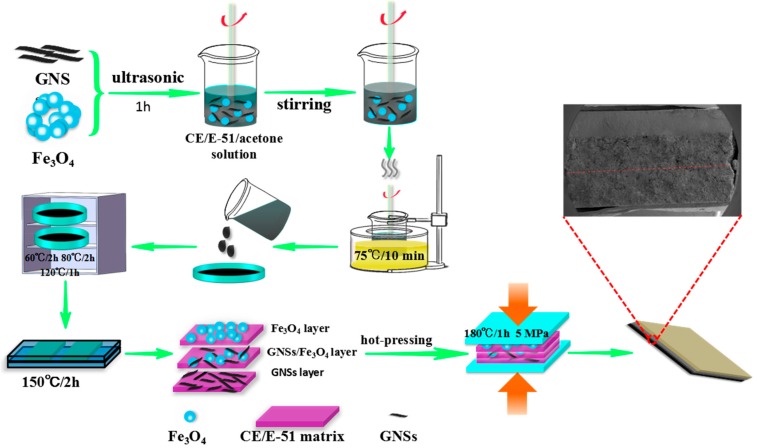
Schematic for the fabrication of layer-structured the GNSs/Fe_3_O_4_/CE composite.

The schematic diagram of the fabrication of the layer-structured GNSs/Fe_3_O_4_/CE nanocomposites is shown in Figure 1. Firstly, GNSs and Fe_3_O_4_ were added to as-prepared CE/E-51/acetone mixed solution (the specific content and ratio are shown in Table S1) and dispersed ultrasonically for 1 h. Then the mixed solution was heated in oil bath pan at 75 °C for 10 min to remove acetone. Thereafter, the mixture was transferred into a glass petri dish and placed in an oven at 60 °C/2 h, 80 °C/2 h, and 120 °C/1 h, respectively, in order to the further remove of acetone (60 °C/2 h) and the prepolymerization of CE (80 °C/2 h and 120 °C/1 h). Subsequently, the prepolymerized GNSs/Fe_3_O_4_/CE was transferred to metallic mold and heated at 150 °C/2 h to obtain the prepolymerized interlayer. According to the same steps, the prepolymerized initial layer and bottom layer were prepared. Upon completion, the initial layer, interlayer, and bottom layer were stacked layer by layer, and hot-pressing at 180 °C/1 h under pressure of 5 MPa to obtain the layer-structured composites. In order to make a contrast, the homogeneous-structured nanocomposites were also prepared by mixing GNSs and Fe_3_O_4_ into CE/E-51/acetone mixed solution according to our previous work [21]. The homogeneous-structured composite was obtained, in which the content of GNSs, Fe_3_O_4_, and thickness were consistent with that of the layer-structured composites. The resulting composites by laminated stacking and uniform mixing method were marked as layer-structured and homogeneous-structured composites, and abbreviated as LGxFy and HGxFy, respectively. L and H stand for homogeneous and layer structure. G and F denote the GNSs and Fe_3_O_4_, respectively. x and y represent the content of GNSs and Fe_3_O_4_ in the composites, respectively. The sample identification is shown in Table 1.

### 2.3. Characterization

Cross-sections for the scanning electron microscopy (SEM) observations were obtained by cryo-fracturing the specimens after rapid immersion in liquid nitrogen for 30 min beforehand. The fractured surfaces were sputter-coated with gold before the observation in a field emission SEM (Model S-450, Hitachi Corporation, Tokyo, Japan) at an accelerating voltage of 20 kV. Magnetic properties of Fe_3_O_4_ nanoparticles were measured at 300 K with a Lake Shore 7410 vibrating sample magnetometer (VSM) (Acros Organics Co., Morris Plains, NJ, USA). Fourier transform infrared (FTIR) spectra for the GNSs were recorded between 500 and 4000 cm^−1^ with a resolution of 2 cm^−1^ on an IS10 Infrared Spectrometer (Birmingham, AL, USA). The electrical conductivity of the composite samples was measured using a Keithley electrometer Model 4200-SCS (San Francisco, CA, USA) according to a two-point method. Before the test, both ends of the rectangular specimens were coated with silver paste to reduce the contact resistance between specimens and electrodes. The electromagnetic parameters of the nanocomposites were obtained using a network analyzer (Agilent Technologies E8362B, Jacksonville, FL, USA) at X-bands (the microwave frequency range of 8.2–12.4 GHz). The samples were cut into rectangular sheet (22.58 mm × 10.14 mm × 2 mm thickness) before measuring. Then the samples were placed in the specimen holder. The scattering parameters (*S*_11_ and *S*_21_) in the frequency range of 8.2–12.4 GHz were recorded to calculate the coefficients of reflectance (*R*), absorbance (*A*) and transmission (*T*), EMI SE (SE_T_), microwave reflection (SE_R_), and microwave absorption (SE_A_) using the following equations:

(1) $$R=|S_{11}{|}^{2},\;T=|S_{21}{|}^{2}$$

(2) A=1−R−T

(3) $${\mathrm{SE}}_{R}\left(\mathrm{dB}\right)=-10\;\log\left(1-R\right),\;{\mathrm{SE}}_{A}\left(\mathrm{dB}\right)=-10\;\log\left(T/\left(1-R\right)\right)$$

(4) $${\mathrm{SE}}_{T}\left(\mathrm{dB}\right)={\mathrm{SE}}_{R}+{\mathrm{SE}}_{A}+{\mathrm{SE}}_{M}$$

where ${\mathrm{SE}}_{M}$ is the microwave multiple internal reflections, which can be negligible when ${\mathrm{SE}}_{T}$ is higher than 10 dB [49].

## 3. Results

### 3.1. Structural Characterization

**Figure 2:**
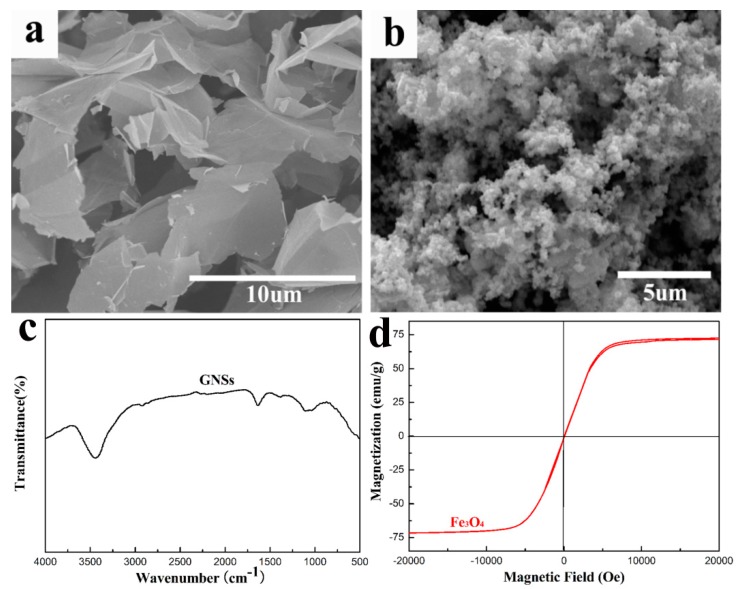
SEM images of the (**a**) GNSs; (**b**) Fe_3_O_4_ nanoparticles; (**c**) FTIR spectra of GNSs; and (**d**) magnetic hysteresis loop of Fe_3_O_4_.

The SEM of the GNSs and Fe_3_O_4_ are shown in Figure 2a,b, respectively. It can be observed clearly that two-dimensional GNSs represent a thin-layered structure, and they interlace with each other. However, the cluster of nano-sized Fe_3_O_4_ particles exhibits significant aggregation due to strong interactions. Figure 2c shows FTIR spectra of the GNSs. Two obvious absorption peaks at around 3421 and 1630 cm^−1^, corresponding to –OH of the adsorbed water and C=C stretching vibration of benzene ring, respectively [50]. The hysteresis loop of Fe_3_O_4_ is shown in Figure 2d. The saturation magnetization (Ms) value of the Fe_3_O_4_ reached 71.5 emu/g, indicating a certain magnetic response to a varying magnetic field [51].

**Figure 3:**
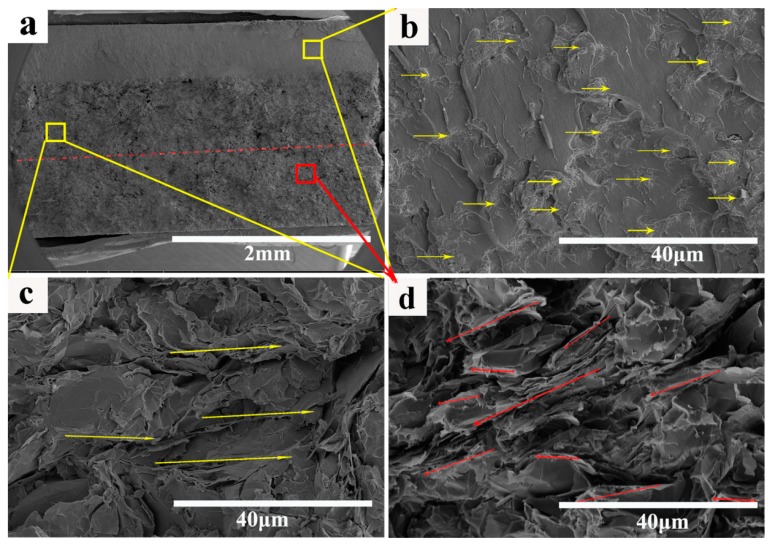
SEM images of the layer-structured nanocomposites (LG5F15): (**a**) low magnification; (**b**–**d**) high magnification of initial layer (Fe_3_O_4_ layer); interlayer (GNSs and Fe_3_O_4_ layer); and bottom layer (GNSs layer); respectively.

The cross-sectional morphology of the layer-structured nanocomposites in Figure 3 reveals three layers distributed in the nanocomposites. The thickness of each layer was approximately 0.7 mm (Figure 3a). Figure 3b–d illustrates the SEM of the initial layer (Fe_3_O_4_ layer), interlayer (GNSs and Fe_3_O_4_ layer), and bottom layer (GNSs layer), respectively. As shown in Figure 3b, there is a serious agglomeration phenomenon of Fe_3_O_4_ in the CE matrix. This is attributed to the fact that Fe_3_O_4_ particles possess small dimension and strong interactions [21,52]. Comparing the SEM of the initial layer, the agglomeration phenomenon disappeared evidently in the interlayer. This is attributed to the lamellar structure of GNSs, which prevent the precipitation and aggregation of high density of Fe_3_O_4_ (Figure 3c). It is clearly seen from Figure 3d that the GNSs form a certain orientation (red arrows). The well-interconnected GNSs are beneficial to achieving a higher conductivity and higher EM wave reflection efficiency, providing the composites with greater EMI SE.

### 3.2. Electrical Property of Nanocomposites

**Figure 4:**
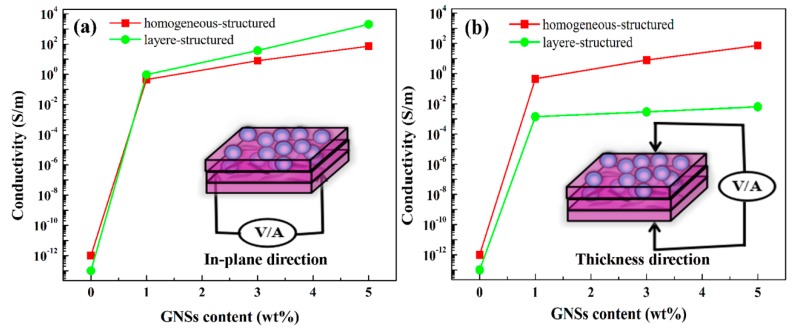
Electrical conductivity of homogeneous-structured and layer-structured composites (**a**) in the in-plane direction and (**b**) thickness direction.

The electrical conductivity of the homogeneous-structured composites and layer-structured composites are depicted in Figure 4. As shown in Figure 4a, the in-plane conductivity mainly refers to the bottom electrical conductivities, and it was found to have increased significantly with the augment of GNSs. In addition, the electrical conductivity of the layer-structured composite was always higher than that of homogeneous-structured composite, especially for high GNSs content. For instance, the maximum electrical conductivity value (up to 2000 S/m) of layer-structured composite (LG5F15) was thirty times higher than that of the homogeneous-structured (72.75 S/m) composite at the same filler content. This is due to the in-plane direction that can be regarded as a parallel circuit, so its resistivity depends largely on the smallest of parallel circuits. Thus, the in-plane conductivity is attributed to the extremely high conductivity of the bottom layer. Figure 4b illustrates the electrical conductivity in the thickness direction. It is interesting to note that the electrical conductivity of the layer-structured composites was four orders of magnitude lower than the electrical conductivity in the in-plane direction. The reason is that the electrical conductivity of the composite in the thickness direction mainly depends on the conductivity of the initial layer (Fe_3_O_4_ layer), and the content of GNSs has little effect on the electrical conductivity in the thickness direction. Thus, the lower electrical conductivity of the initial layer, the more electromagnetic microwaves enter into the composite and endow improved absorption rather than the reflection of electromagnetic waves.

### 3.3. EMI Shielding Performance of Nanocomposite

**Figure 5:**
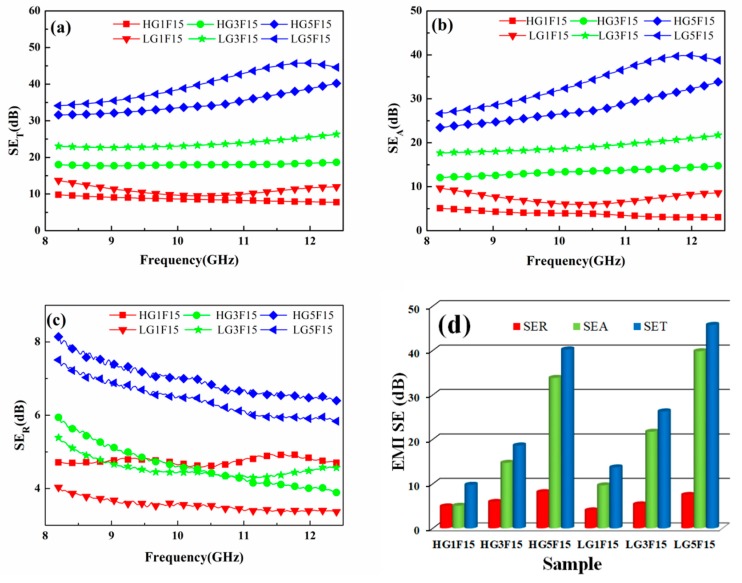
EMI SE of the nanocomposites: (**a**) SE_T_; (**b**) SE_A_; and (**c**) SE_R_ of the nanocomposites in the X-band; (**d**) the comparison of SE_T_, SE_A_, and SE_R_ for the nanocomposites with different filler contents and structures at the frequency of 12 GHz.

The EMI SE values against the X-band frequency range (8.2–12.4 GHz) for nanocomposites with homogeneous and layered structure are displayed in Figure 5, and the electromagnetic parameters and SE_T_ of CE are shown in Figure S1. Both the homogeneous and layered samples exhibit frequency-independent EMI SE values over the measured frequency range, as shown in Figure 5. In addition, the EMI SE values improve with the GNSs contents increasing. In particular, compared with the homogeneous-structure nanocomposites, the composites with the layer-structured show superior EMI SE. When the content of GNSs is 5 wt % and Fe_3_O_4_ is 15 wt %, the EMI SE values of layer-structured nanocomposites with a thickness of 2.0 mm is 45.7 dB, far exceeding the homogeneous-structure nanocomposites (39.8 dB). This implied that the EMI SE was enhanced after optimizing the distribution of the fillers in the nanocomposites.

Figure 5b shows the SE_A_ values. It is worth noting that the SE_A_ values of layer-structured are always higher than that of homogeneous-structured nanocomposites under the same filler content. At the same time, from Figure 5c, it is obvious that the layer-structured nanocomposites exhibit lower SE_R_ values comparing to the filler distributed homogeneously nanocomposites. In addition, the comparison of SE_T_, SE_A_, and SE_R_ for the two type composites at the frequency of 12.0 GHz (Figure 5d) indicates that the increased SE_T_ of the composite was mainly based on the improved absorption rather than the reflection of electromagnetic waves.

**Figure 6:**
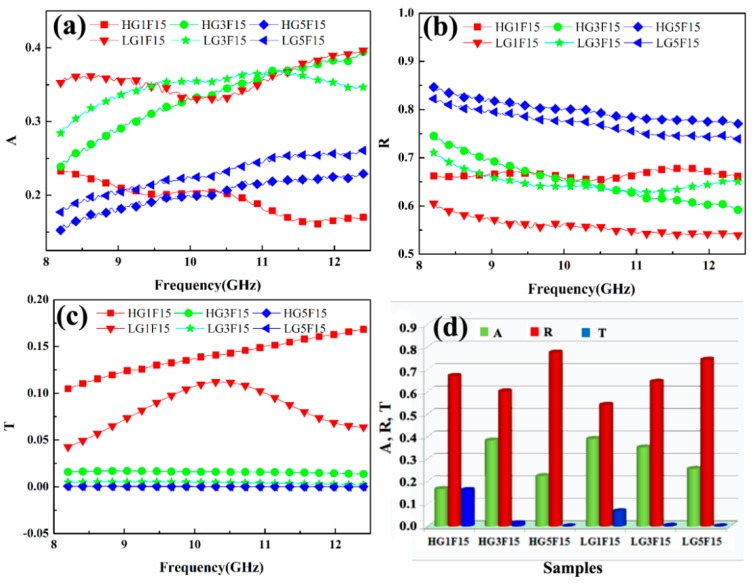
*A*, *R*, and *T* values of the nanocomposite (**a**) *A*; (**b**) *R*; and (**c**) *T* in the X-band; (**d**) the comparison of *A*, *R*, and *T* for the nanocomposites with different filler contents and structures at the frequency of 12 GHz.

Generally, EMI shielding performance of a material can be described by coefficients of reflectance (*R*), absorbance (*A*), and transmission (*T*), which were calculated from magnitudes of S-parameters *S*_11_ and *S*_21_. They represent the capability of a material to reflect, absorb, and transmit the microwave, respectively. The *R*, *A*, and *T* values of two kinds of nanocomposites with different structures are depicted in Figure 6. It can be seen that the *R* value is higher than the *A* value for the nanocomposite with the same structure, which indicates that the reflection mechanism holds the dominant factor. This is due to the high electrical conductivity of the nanocomposites. Compared with the homogeneous-structured nanocomposites, the *A* values of the layer-structured nanocomposites are higher than homogeneous-structured nanocomposites, indicating that absorption-dominated shielding occurs when constructing gradient shielding layers. This is due to that the first layer (magnetic granular layer) of the layer-structured nanocomposite is good for enhancing impedance matching so that more microwaves enter the nanocomposite, which are attenuated by coherent multiple reflections at the internal interfaces of conductive and magnetic particles layers. Besides, *T* values of the nanocomposite decreases from 0.17 to ~10^−4^ of homogeneous-structured nanocomposite, and 0.11 to ~10^−5^ of layer-structured nanocomposite, indicating that layer-structured nanocomposite has a better shielding effect.

The electromagnetic properties of a material, namely electrical permittivity and magnetic permeability, determine its response to the electromagnetic waves. Permittivity and permeability are the complex numbers and determine how the material interacts with the electrical and magnetic fields of the wave, respectively. The corresponding results and analysis are shown in Figure S3. More importantly, on the other side (apply the incident wave from the bottom layer of the composite), we also tested the scattering parameters (*S*_11_ and *S*_21_) in the frequency range of 8.2–12.4 GHz from a different side of the sample and calculated the EMI SE (SE_T_), microwave reflection (SE_R_), and microwave absorption (SE_A_), and the corresponding results and analysis are shown in Figure S2.

### 3.4. Theoretical Simulation

**Figure 7:**
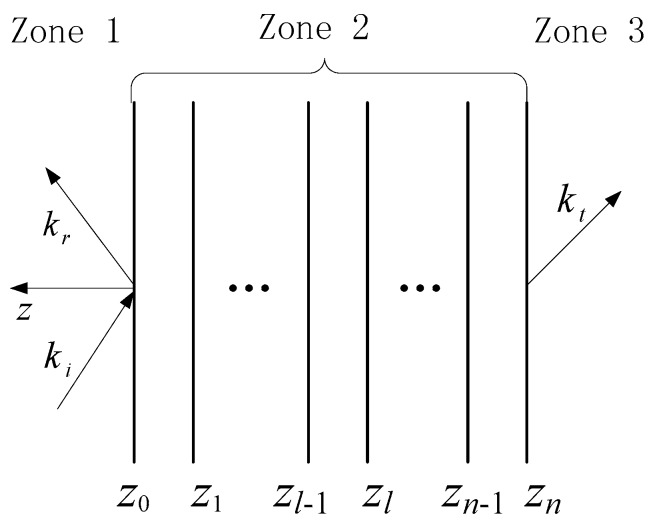
Wave propagation in a multilayer structure.

To verify the effectiveness of our design and provide theoretical tools for further development, we built a simulated process based on the generalized propagation matrix method. Figure 7 illustrates the wave propagation in a multilayer structure, where *k_i_*, *k_r_*, and *k_t_* are the wave vector for the incident wave, reflected wave, and transmitted wave, zone 2 is the layered structure, and zone 1 and zone 3 are the free space. The interfaces of the different layers are perpendicular to the z-axis, and the top and bottom layers are located in the *z* = *z*_0_ plane and *z* = *z_n_* plane, respectively.

Then, we have

(5) $$\left[\begin{array}{c} 0\\ T \end{array}\;\right]=A_{3}^{-1}\cdot P_{2}\left(z_{n},z_{0}\right)\cdot A_{1}\cdot \left[\begin{array}{c} R\\ I \end{array}\right]$$

where *T* and *R* are the 2 × 2 transmission coefficient matrix and reflection coefficient matrix,

(6) $$R=\left[\begin{array}{cc} R_{11\;} & R_{12}\\ R_{21} & R_{22} \end{array}\right]$$

(7) $$T=\left[\begin{array}{cc} T_{11\;} & T_{12}\\ T_{21} & T_{22} \end{array}\right]$$

and *I* is the identity matrix, *P*_2_ is the propagation matrix for zone 2, *A*_1_ and *A*_3_ are the eigenvector matrixes for zone 1 and zone 3, respectively. More details about this method can be found in References [25,53,54].

**Figure 8:**
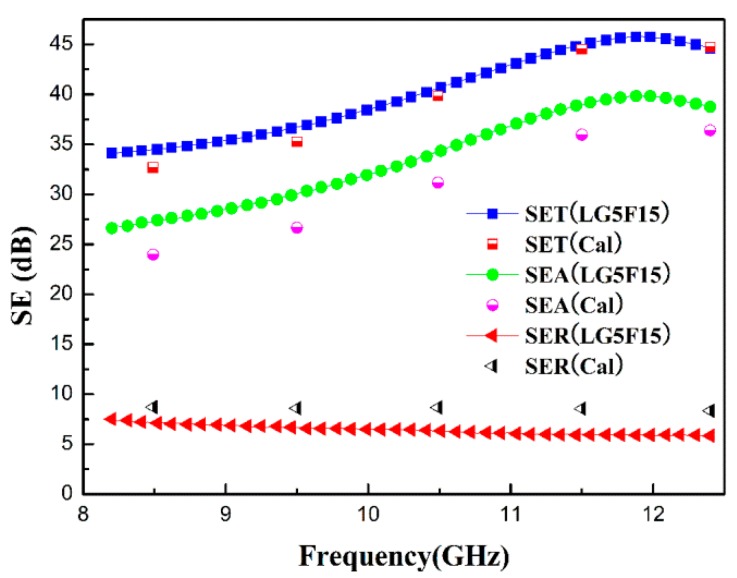
Comparison between calculated and measured SE_R_, SE_A_, and SE_T_ for LG5F15.

Finally, the electromagnetic parameters for the three different layers of LG5F15, i.e., top layer with 30 wt % Fe_3_O_4_, middle layer with 5 wt % GNS and 15 wt % Fe_3_O_4_, and bottom layer with 10 wt % GNS, are utilized to calculate the SE_R_, SE_A_, and SE_T_. The total thickness is 2 mm and all layers have the same thickness with each other. The obtained results are shown in Figure 8.

According to Figure 8, the calculated EMI SE values are in good agreement with the measured curves, which verifies the effectiveness of our layered strategy and the correctness of the theoretical model. The difference is mainly due to the unavoidable error in obtaining the electromagnetic parameters for different layers. This model will be a useful theoretical tool to further develop our layered nanocomposite.

## 4. Conclusions 

In summary, layer-structured CE nanocomposites with efficient EMI shielding performance were fabricated from GNSs, Fe_3_O_4_, and CE via mechanical mixing and hot-pressing method. By employing electrical and magnetic loss fillers and adjusting its distribution, the resultant multilayered structures were endowed with substantial improvements in overall shielding effectiveness. The layer-structured PCs with very low nanofiller content (5 wt % GNSs and 15 wt % Fe_3_O_4_) and a thickness of only 2 mm exhibited ultrahigh electrical conductivity and excellent electromagnetic interference shielding effectiveness (EMI SE), reaching up to 2000 S/m and 45.7 dB in the X-band, respectively. The upper surface (Fe_3_O_4_ layer) allows more electromagnetic waves to penetrate into the composite, and the interlayer endows the composite to have strong absorption ability because of the significant magnetic hysteresis loss of Fe_3_O_4_ and dielectric loss of GNSs, while the dense GNS network deposited at the bottom of the composite provides an excellent electromagnetic shielding ability because of its remarkable conductivity. This specific structure results in the “absorb-reflect-reabsorb” process for the incident electromagnetic waves, leading to an excellent EMI shielding performance. This novel layer-structured shielding network design can serve as a strategy for designing highly efficient EMI shielding materials with low reflection characteristics.

## Figures and Tables

**Table 1:** Sample identification of composites.

Sample Name	Structures	Total Fillers	Fillers Distribution in Each Layer	Thickness
HG1F15	Homogeneous-structured	1 wt % GNSs + 15 wt % Fe_3_O_4_	~	2 mm
HG3F15		3 wt % GNSs + 15 wt % Fe_3_O_4_	~	
HG5F15		5 wt % GNSs + 15 wt % Fe_3_O_4_	~	
LG1F15	Layer-structured	1 wt % GNSs + 15 wt % Fe_3_O_4_	Initial: 30 wt % Fe_3_O_4_	
			Interlayer: 1 wt % GNSs + 15 wt % Fe_3_O_4_	
			Bottom: 2 wt % GNSs	
LG3F15		3 wt % GNSs + 15 wt % Fe_3_O_4_	Initial: 30 wt % Fe_3_O_4_	
			Interlayer: 3 wt % GNSs + 15 wt % Fe_3_O_4_	
			Bottom: 6 wt % GNSs	
LG5F15		5 wt % GNSs + 15 wt % Fe_3_O_4_	Initial: 30 wt % Fe_3_O_4_	
			Interlayer: 5 wt % GNSs + 15 wt % Fe_3_O_4_	
			Bottom: 10 wt % GNSs	

## Supplementary Materials

The following are available online at http://www.mdpi.com/2073-4360/10/9/933/s1, Figure S1: (**a**) The electromagnetic parameters of CE and (**b**) the SE_T_ of CE. Figure S2: EMI SE of the nanocomposites (apply the incident wave from the bottom layer of the composite): (**a**) SE_T_; (**b**) SE_A_; and (**c**) SE_R_ of the nanocomposites in the X-band; (**d**) The comparison of SE_T_, SE_A_, SE_R_ for the nanocomposites with different filler contents and structures at the frequency of 12 GHz. Figure S3: Electromagnetic characteristics of the composites in the range of 8.2–12.4 GHz: (**a**) real and (**b**) imaginary parts of the electrical permittivity; (**c**) real and (**d**) imaginary parts of the magnetic permeability. Table S1: The composition and specific content of the composite.
