# Supplementary material for: Layer-Structured Design and Fabrication of Cyanate Ester Nanocomposites for Excellent Electromagnetic Shielding with Absorption-Dominated Characteristic
Source: Polymers (Basel). 2018 Aug 21;10(9):933. doi: 10.3390/polym10090933 (PMC6404011; doi:10.3390/polym10090933)
Supplement: Supplementary file 1 [file polymers-10-00933-s001.pdf]

Supplementary Materials: Layer-Structured Design and Fabrication of Cyanate Ester Nanocomposites for Excellent Electromagnetic Shielding with Absorption-Dominated Characteristic

Fang Ren, Zheng-Zheng Guo, Han Guo, Li-Chuan Jia, Yu-Chen Zhao, Peng-Gang Ren and Ding-Xiang Yan

The composition and specific content of the composite were shown in Table S1.

Table S1. The composition and specific content of the composite.

Sample Name	Structures		Matrix		Fillers	
			CE (g)	E-51 (g)	GNSs (g)	Fe_3O_4 (g)
HG1F15	Homogeneous-structured		2.4	0.6	0.036	0.536
HG3F15	Homogeneous-structured		2.4	0.6	0.110	0.549
HG5F15	Homogeneous-structured		2.4	0.6	0.188	0.562
LG1F15	Layer-structured	Initial	0.8	0.2	0	0.357
		Interlayer	0.8	0.2	0.012	0.179
		Bottom	0.8	0.2	0.024	0
LG3F15	Layer-structured	Initial	0.8	0.2	0	0.366
		Interlayer	0.8	0.2	0.037	0.183
		Bottom	0.8	0.2	0.073	0
LG5F15	Layer-structured	Initial	0.8	0.2	0	0.375
		Interlayer	0.8	0.2	0.063	0.187
		Bottom	0.8	0.2	0.125	0

The electromagnetic parameters and SE_T of CE have been shown in Figure S1. The real (ε') and imaginary (ε'') part of the dielectric constant of the CE, which showed in Figure S1(a), were found to be nearly constant around, $\varepsilon' = 2.9$ and $\varepsilon'' = 0.05$ over entire frequency range 2–18 GHz. From Figure S1 (b), we can clearly see that SE_T is almost zero, indicating the CE has little electromagnetic shielding performance.

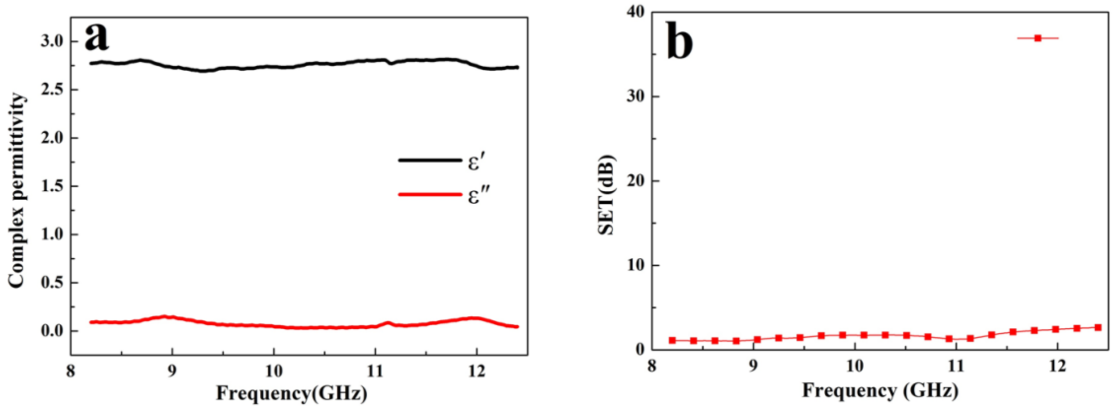

Figure S1. (a) The electromagnetic parameters of CE and (b) the SE_T of CE.

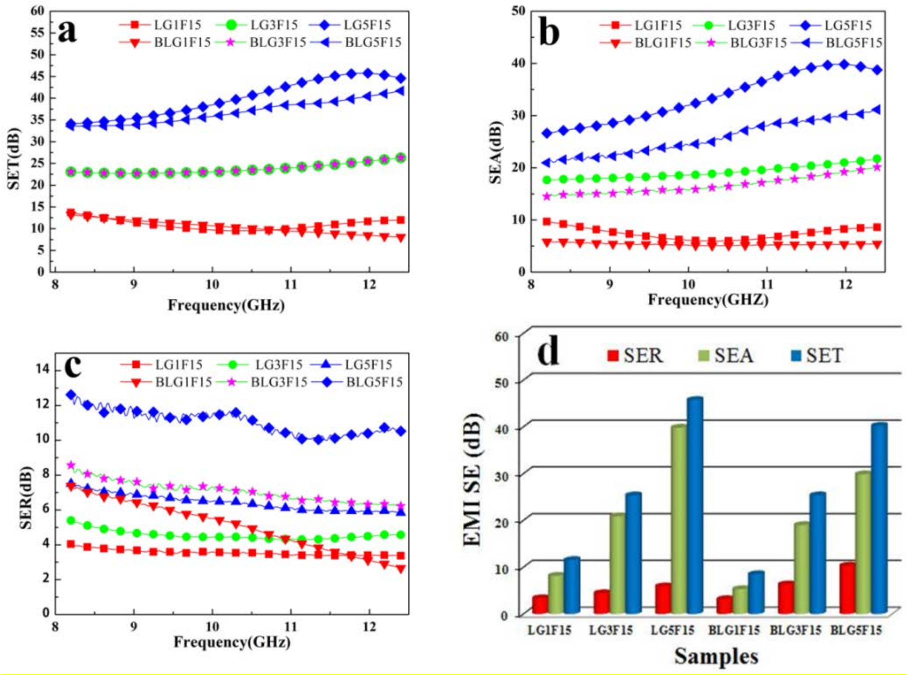

Figure S2. EMI SE of the nanocomposites (apply the incident wave from the bottom layer of the composite): (**a**) SE_T; (**b**) SE_A; and (**c**) SE_R of the nanocomposites in the X-band; (**d**) The comparison of SE_T, SE_A, SE_R for the nanocomposites with different filler contents and structures at the frequency of 12 GHz.

BLG1F15, BLG3F15 and BLG5F15 represent electromagnetic waves coming from the bottom during testing. From Figure S2, SE_T of the all composites increase as the filler content increased, at the same time, they are nearly the same at equal amounts of filler content. This result illustrates that the incident direction has no clear effect on the SE_T of the composites. However, the SE_R of the composites show a huge difference at equal amounts of filler content. Applying the incident wave from the bottom layer of the composite get higher SE_R and lower SE_A than applying the incident wave from the initial layer of the composite, which is attributed to bottom layer possesses high electricity conductivity, causing the impedance mismatch between the surface of the composites and the electromagnetic wave. These results indicate that the layered structure design can significantly attenuate backscattered signals.

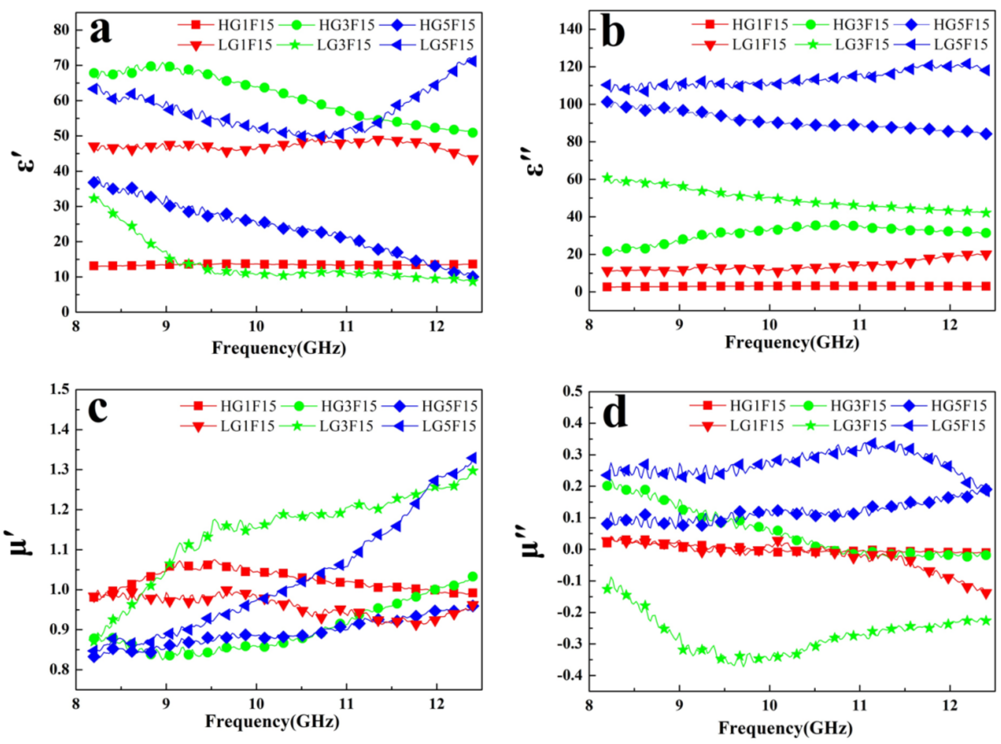

Figure S3. Electromagnetic characteristics of the composites in the range of 8.2–12.4 GHz: (**a**) real and (**b**) imaginary parts of the electrical permittivity; (**c**) real and (**d**) imaginary parts of the magnetic permeability.

Figure S3 shows the electrical permittivity ($\varepsilon = \varepsilon' - j\varepsilon''$) and magnetic permeability ($\mu = \mu' - j\mu''$) of the homogeneous-structured and layer-structured composites in the frequency range of 8.2–12.4 GHz. As shown in Figure S3 a–b, the ε' of HG1F15 is a little higher than 10 and its ε'' is close to zero, indicating the weak dielectric loss of the composite. The ε' and ε'' increased significantly with the increase of GNSs content. For instance, the ε' and ε'' of HG5F15 are in the ranges of 10.1–36.8 and 84.3–101.4, respectively, which are much higher than those of HG1F15. The ε' of the composite are determined by the configuration and internal fractal structure. The higher content of two-dimensional GNSs incorporates into the resin, the more GNSs/Fe_3O_4/resin interfaces are formed, which certainly increases the ε' of such composites. More importantly, as shown in Figure S3b, the ε'' not only obviously increases with the increase of GNSs content, but also layer-structured composites are higher than homogeneous-structured composites under the same composition, which is attributed to the high electrical conductivity and interconnected network of GNSs.

Comparison of μ' and μ'' of homogeneous-structured and layer-structured, the layer-structured composite exhibits higher values of μ' and μ'', as shown in Figure S3 c–d. It is reasonable that the layer-structured composites possess higher values of μ' and μ'' because their surface layers have more magnetic fillers, which is related to the magnetic permeability of the magnetic particle-filled composites.
